# Severe Mycobacterium chelonae Infection Following Dermal Filler Injection: A Diagnostic and Therapeutic Challenge

**DOI:** 10.7759/cureus.79784

**Published:** 2025-02-27

**Authors:** Rute Aleixo, Magda Gonçalves, Rosa Sá, Isabel Ramos, Cristina Valente

**Affiliations:** 1 Infectious Diseases, Unidade Local de Saúde de Coimbra, Coimbra, PRT

**Keywords:** atypical mycobacterial infection, cosmetic procedure complication, dermal filler infection, invasive surgical debridement, multidisciplinary management, mycobacterium chelonae, non-tuberculous mycobacteria, skin and soft tissue infection

## Abstract

This case report describes a challenging case of *Mycobacterium chelonae* infection in a 72-year-old immunocompetent woman following poly-L-lactic acid injection. This represents the first reported case of *Mycobacterium chelonae* ​​​​infection following dermal filler injections in Europe. Despite initial empiric therapy, the infection progressed, requiring multiple surgical debridements and prolonged antibiotic therapy with significant side effects. This report highlights the challenges of diagnosing and treating *Mycobacterium chelonae* infections, emphasizing the importance of early recognition, a multidisciplinary approach, and susceptibility-guided antibiotic therapy. The gravity of this case underscores the potential for severe complications following seemingly minor cosmetic procedures, even in immunocompetent individuals. It highlights the need for a high index of suspicion and prompt medical attention.

## Introduction

*Mycobacterium chelonae* is a rapidly growing, nontuberculous mycobacterium (NTM) commonly found in environmental sources such as water and soil. It can also be associated with plants, fish, and free-living amoebae [1,2]. While typically considered an opportunistic pathogen, *Mycobacterium chelonae* can cause localized infections, particularly following traumatic inoculation [2,3]. These infections range from mild skin lesions to more severe, disseminated disease, especially in immunocompromised individuals. These infections can be particularly challenging to diagnose and treat due to their rarity, potential for delayed presentation, and frequent resistance to conventional antibiotics [4].

Cosmetic interventions, particularly facial aesthetic procedures like dermal fillers, have gained significant popularity among individuals seeking non-invasive enhancements to their appearance. Poly-L-lactic acid is a synthetic, biodegradable polymer used in cosmetic procedures to stimulate collagen production and improve facial volume. While adverse events are infrequent, complications may occur [5,6]. Infections, particularly those caused by NTM, are rare but potentially serious adverse events following dermal filler injections [3].

*Mycobacterium chelonae* infections have been increasingly reported in recent decades, particularly following medical and surgical procedures, including cosmetic interventions such as facelifts, breast surgery, and liposuction, likely due to inadequate sterilization of surgical instruments. Furthermore, due to contaminated materials, they have also been associated with mesotherapy, acupuncture, tattooing, and nail salon visits [1,2,7]. Tap water exposure is the primary risk factor for healthcare-associated infections [2,7]. Interestingly, *Mycobacterium chelonae* infections have also been linked to medical tourism, suggesting a potential role of substandard healthcare practices in certain regions [7,8]. While disseminated cases are more common in immunocompromised individuals [1,7], this case report presents a unique and challenging clinical course of *Mycobacterium chelonae* infection in an immunocompetent patient after injection of poly-L-lactic acid. After a thorough literature review, this is the first reported case of *Mycobacterium chelonae* infection following dermal filler injections in Europe. The patient experienced severe and extensive facial and neck lesions, requiring multiple surgical debridements and prolonged antibiotic therapy with significant side effects. The diagnostic delay, the severity of the infection, and the management complexities encountered in this case underscore the importance of considering NTM infections in the differential diagnosis of post-injection complications and highlight the need for a high index of suspicion, prompt diagnosis, and aggressive management.

## Case presentation

A 72-year-old female patient with no significant medical history or known immunosuppressive factors underwent a cosmetic procedure involving the injection of poly-L-lactic acid in the face and neck in August 2022. Shortly after, the patient began to develop inflammatory signs on the treated region and sought medical attention from her general practitioner. She was diagnosed with cellulitis and empirically treated with ciprofloxacin 750 mg every 24 hours, which resulted in minimal improvement. Upon reassessment, metronidazole 500 mg every 24 hours was prescribed. Initially, her condition showed slight improvement, but it gradually worsened thereafter.

In December 2022, the patient presented to the emergency department with lesions on the face and neck that persisted for several weeks. These were erythematous papules and nodules in different stages, as well as non-healing ulcers with irregular margins, with areas of necrosis and seropurulent drainage. The area was tender and deeply painful. She had no systemic symptoms, and there were no other abnormalities during the physical examination. Laboratory results were unremarkable. The dermatology and maxillofacial surgery departments assessed her, leading to a probable diagnosis of a foreign body reaction. Treatment with amoxicillin 1000 mg/clavulanic acid 200 mg and dexamethasone 5 mg every eight hours was initiated, and the patient was admitted to the maxillofacial surgery department, where an initial exploration and surgical debridement were performed, excising nodular indurated lesions and fibrotic areas. Samples were collected only for routine bacterial and fungal cultures, all yielding negative results. After each debridement or drainage procedure, the drainage fluid, pus, and biopsy samples were sent to the microbiology laboratory for culture analysis.

**Figure 1:**
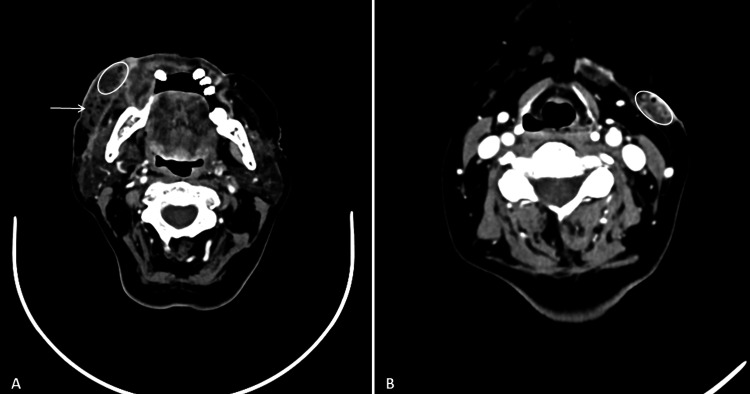
Axial contrast-enhanced CT scans: (A) The arrow indicates subcutaneous thickening and heterogeneity of the right hemiface with areas of subcutaneous gas. The oval contour highlights a hypodense collection with an internal gas bubble measuring 8.7 mm. (B) The oval contour marks a hypodense lesion within the hypodermis at the transition between the left submandibular and left cervical regions, containing small gas bubbles and measuring 17 × 8 mm, consistent with an abscess. CT: computed tomography

The patient continued treatment with amoxicillin/clavulanic acid and corticosteroids. However, her lesions worsened. A CT scan revealed subcutaneous thickening and heterogeneity of the right hemiface with air bubbles, an irregular skin surface, and a hypodense collection with a gas bubble. Similar findings were noted on the left hemiface, though less pronounced. No other abnormal collections were identified. An abscess was also described in the left cervical region (Figure 1). Given the lack of clinical improvement, the ongoing antibiotic therapy was discontinued, and empirical treatment with piperacillin 4000 mg/tazobactam 500 mg every six hours and vancomycin 1250 mg every eight hours was initiated.

**Figure 2:**
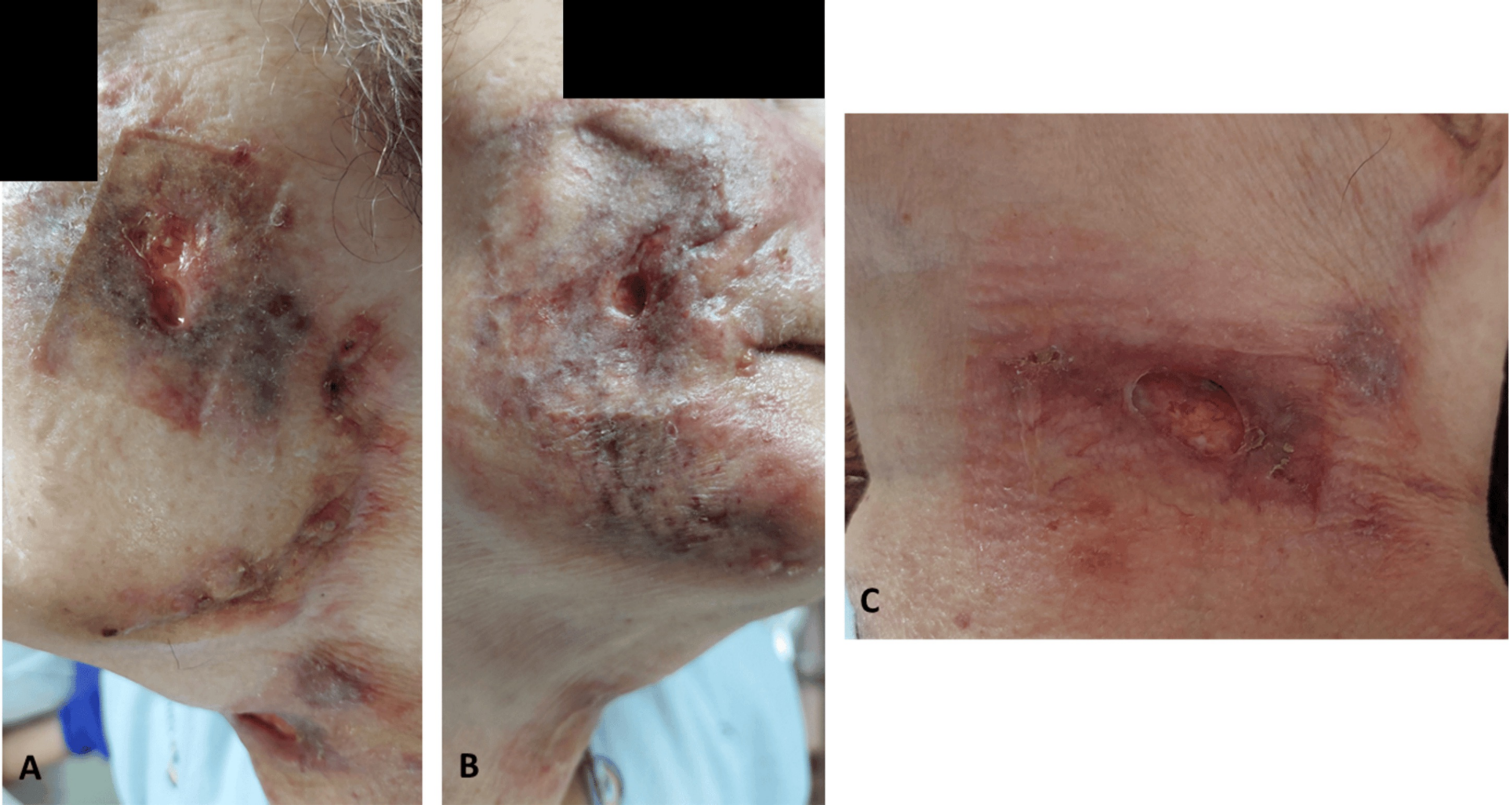
Skin lesions when the patient was admitted to the infectious diseases department: (A) left side of the face, (B) right side of the face, and (C) neck

By late December 2022, the patient had undergone a second surgical debridement, during which the first microbiological investigation for mycobacteria was performed. Direct smear microscopy using Ziehl-Neelsen staining revealed a strongly positive result for acid-fast bacilli, indicating the presence of mycobacteria. Cultures were set up on solid (Löwenstein-Jensen agar) and liquid (mycobacteria growth indicator tube) media and incubated at 30°C and 37°C. Colonies emerged within five days, although the specific pathogen had yet to be identified. Subsequently, the dermatology department collected pus samples for repeat mycobacterial testing and performed a skin biopsy for histopathological analysis. In early January 2023, results from the skin biopsy were delivered, showing a multifocal suppurative granulomatous infiltrate containing acid-fast extracellular bacilli, consistent with atypical mycobacteriosis. Consequently, the patient was transferred to the infectious diseases department, as she continued to present necrotic areas, nodules, ulcers, and seropurulent drainage, despite ongoing treatment (Figure 2).

In mid-January 2023, *Mycobacterium chelonae* was identified for the first time through molecular testing of a previously positive culture. A polymerase chain reaction followed by nucleic acid hybridization confirmed the result, leading to the final diagnosis of skin and soft tissue infection by *Mycobacterium chelonae*. Samples were sent to the national reference laboratory for antimicrobial susceptibility testing. Consequently, antibiotic therapy was switched to clarithromycin 500 mg every 12 hours, imipenem 750 mg every 12 hours, and amikacin 750 mg every 24 hours. The amikacin dose was calculated based on a regimen of 10-15 mg/kg, considering the patient's weight of 58 kg. Renal function was assessed using the CKD-EPI equation, yielding an estimated creatinine clearance of 70 mL/min/1.73m². Therapeutic drug monitoring was performed throughout the hospitalization following standard recommendations, with dose adjustments made as necessary to optimize efficacy and minimize toxicity.

To minimize nephrotoxicity risk, adequate hydration was ensured, guaranteeing adequate fluid intake and electrolyte balance. Electrolyte levels were monitored regularly, and corrections were made as needed. Renal function was closely monitored with serial serum creatinine measurements and estimated creatinine clearance. Additionally, drug scheduling was optimized to minimize renal burden, and a staggered dosing strategy was implemented to further minimize renal toxicity.

**Figure 3:**
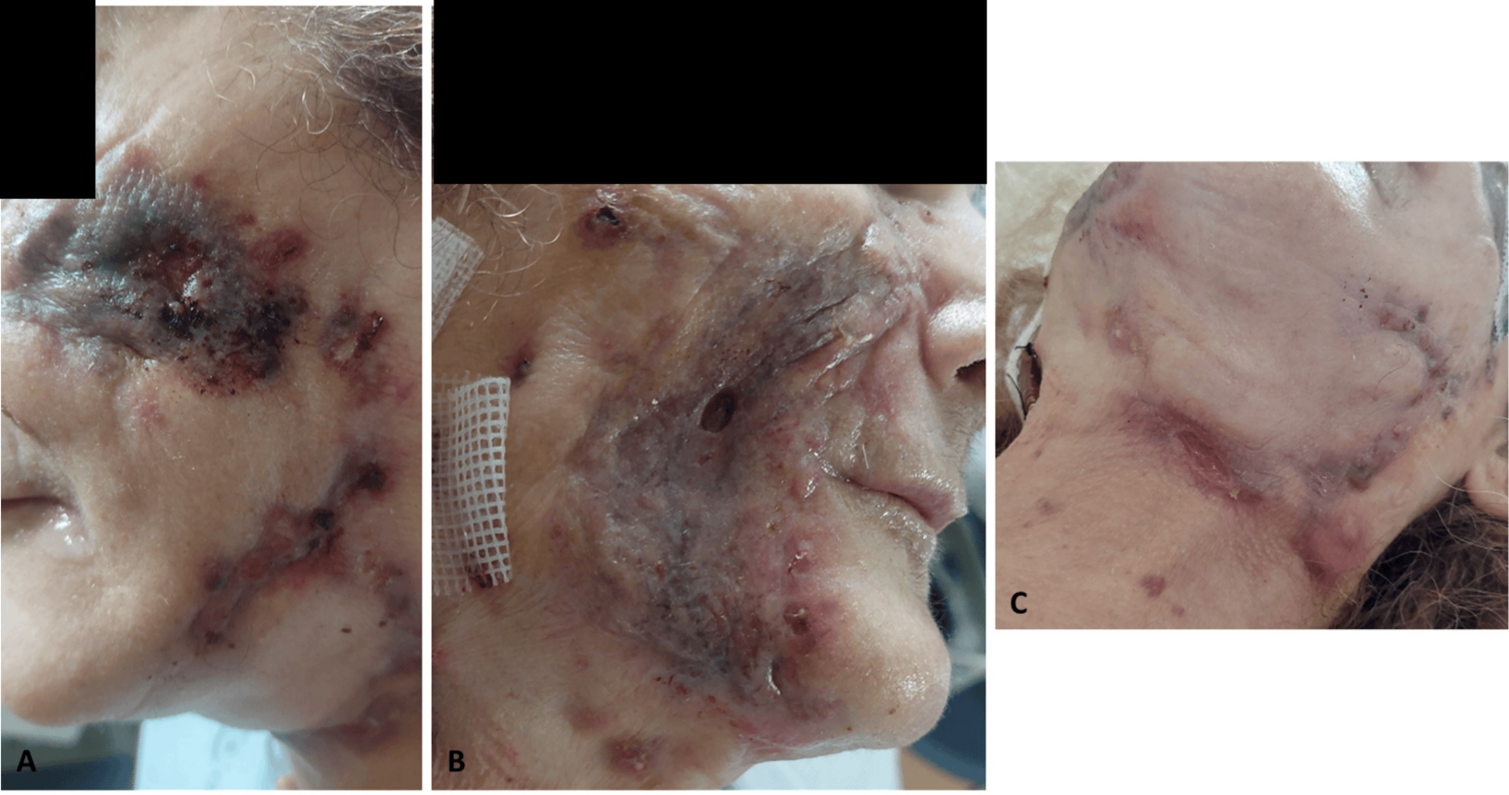
Skin lesions during the period of most severe disease progression: (A) left side of the face, (B) right side of the face, and (C) neck

**Figure 4:**
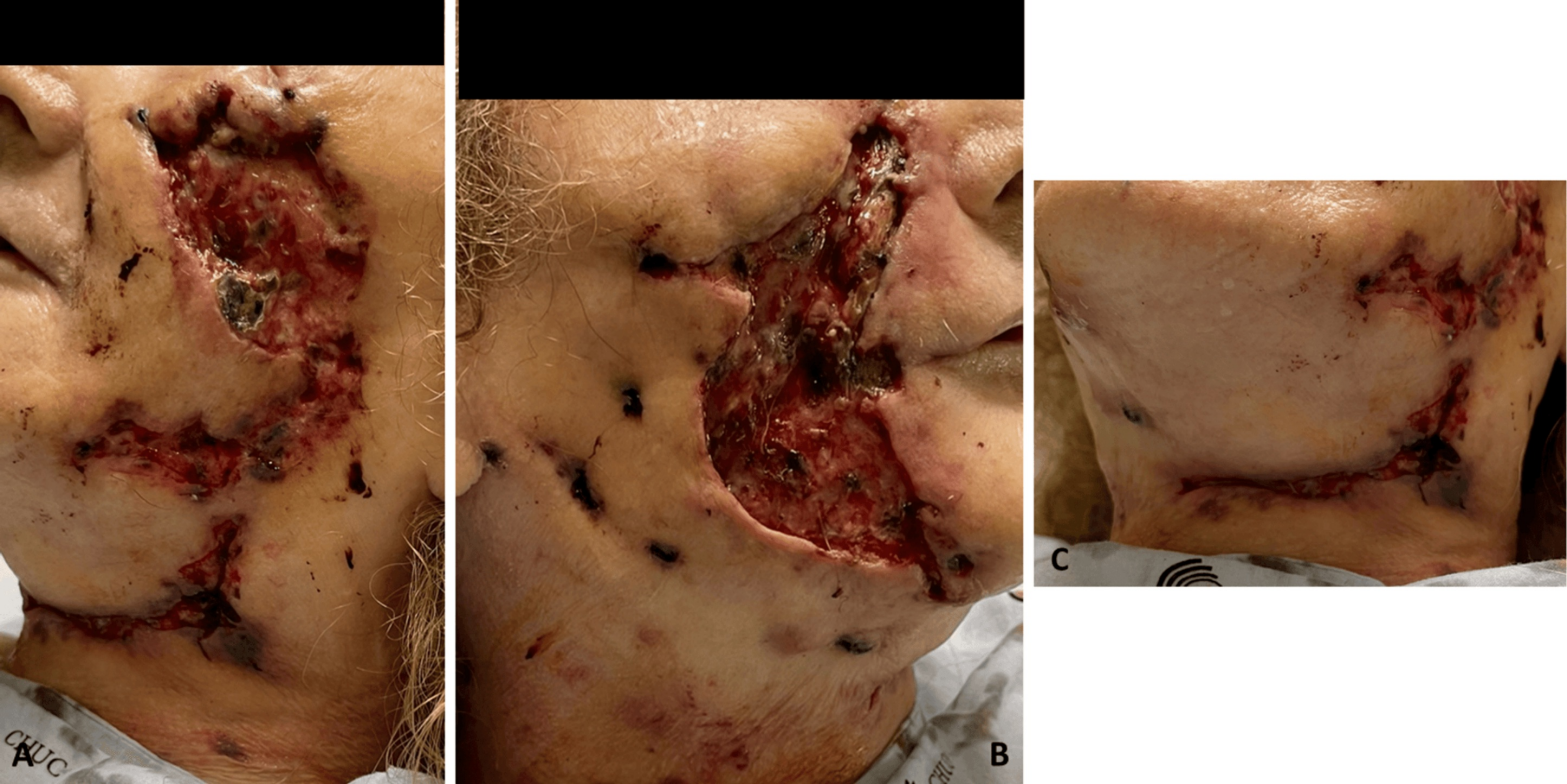
Skin lesions following extensive surgical debridement: (A) left side of the face, (B) right side of the face, and (C) neck

Further microbiological testing of samples collected in January showed strongly positive acid-fast smears and cultures positive for mycobacteria. Despite the patient's initial improvement, her lesions worsened again in mid-February 2023. Due to the extensive and severe nature of the lesions (Figure 3), another surgical debridement was performed in late February 2023, this time being highly invasive (Figure 4) with excision of necrotic skin and subcutaneous tissue while also revealing diffuse fibrosis, particularly in the nasogenian sulci bilaterally. In the right malar region, debridement was extended to the fascial plane, stopping upon reaching the vicinity of the oral cavity.

Following this procedure, direct examination of new samples showed weakly positive acid-fast smears, while cultures were negative for mycobacteria.

In early March 2023, antimicrobial susceptibility testing results were received, showing resistance to sulfamethoxazole, susceptibility with increased exposure to ciprofloxacin, and susceptibility to clarithromycin, amikacin, linezolid, and moxifloxacin. Following surgical focus control, the patient exhibited sustained clinical improvement.

Prolonged antibiotic treatment resulted in several side effects for the patient, who experienced nausea and vomiting associated with the perfusion of imipenem, which was then switched to linezolid 600 mg every 24 hours.

Amikacin led to ototoxicity and nephrotoxicity, significantly impairing the patient's hearing and leading to acute renal failure. Ototoxicity was evaluated upon the onset of hearing impairment. An otolaryngology assessment conducted in mid-April reported moderate sensorineural hearing loss, suspected to be due to either presbycusis or aminoglycoside-induced ototoxicity. However, a definitive attribution could not be made without baseline audiometric data.

The patient also referred palpitations, hypotension, and syncope episodes. A comprehensive cardiac evaluation, including ECG and Holter monitoring, revealed frequent supraventricular and ventricular extrasystoles and tachyarrhythmia episodes. The cardiology department recommended initiating bisoprolol, and after multidisciplinary discussion, these episodes were attributed to a hypotensive profile secondary to drug toxicity, particularly clarithromycin. Given the observed adverse effects, the antibiotic regimen was modified, and clarithromycin was discontinued.

The patient was hospitalized for 155 days, undergoing three surgical debridements. Throughout the hospitalization, multiple additional samples were obtained during subsequent debridements and routine wound care, all remaining negative for aerobes, anaerobes, and fungi.

Prolonged antibiotic therapy was administered, including 107 days of clarithromycin, 39 days of imipenem (discontinued in early March due to nausea and vomiting), 84 days of amikacin (discontinued in mid-April due to ototoxicity and nephrotoxicity), and 57 days of linezolid (initiated after stopping imipenem). Given the prolonged treatment duration and the complexity of managing drug-related toxicities, Table 1 summarizes the antibiotic regimens, detailing dosage, duration, adverse effects encountered, mitigation strategies, and reasons for treatment modifications.

**Table 1:** Summary of antibiotic regimens, associated toxicities, and mitigation strategies ECG: electrocardiogram

Antibiotic	Dosage	Duration	Associated toxicity	Mitigation strategies	Reason for discontinuation
Ciprofloxacin	750mg q24h	Nov, 2022	None reported	—	Empirical treatment discontinued due to lack of improvement
Metronidazole	500mg q24h	Nov, 2022	None reported	—	Empirical treatment discontinued due to lack of improvement
Amoxicillin/clavulanic acid	1000mg/200mg q8h	Dec 11–20, 2022 (10 days)	None reported	—	Stopped due to clinical worsening. Switch to piperacillin/tazobactam and vancomycin
Piperacillin/tazobactam	4000mg/500mg q6h	Dec 20, 2022–Jan 13, 2023 (24 days)	None reported	—	Lack of clinical response
Vancomycin	1250mg q8h	Dec 20, 2022–Jan 13, 2023 (24 days)	None reported	Therapeutic drug monitoring	Lack of clinical response
Imipenem	750mg q12h	Jan 13–Mar 2, 2023 (39 days)	Nausea, vomiting	Discontinuation upon persistent intolerance	Discontinued due to gastrointestinal intolerance
Amikacin	750mg q24h	Jan 13–Apr 17, 2023 (84 days)	Ototoxicity, nephrotoxicity	Therapeutic drug monitoring. Hydration, electrolyte monitoring, renal function monitoring, staggered dosing. Discontinuation upon toxicity	Discontinued due to ototoxicity/nephrotoxicity. Switch to levofloxacin
Clarithromycin	500mg q12h	Jan 13–May 8, 2023 (107 days)	Cardiotoxicity	Cardiac monitoring (ECG, Holter), bisoprolol initiation. Discontinuation upon toxicity	Discontinued due to cardiotoxicity. Switch to doxycycline
Linezolid	600mg q24h	Mar 2–May 2, 2023 (57 days)	Hematologic toxicity	Therapeutic drug monitoring. Blood count monitoring	Discontinued due to hematologic toxicity
Levofloxacin	500mg q24h	Apr 20–Aug 30, 2023 (133 days)	None reported	—	Completed planned course
Doxycycline	100mg q12h	May 8–Aug 30, 2023 (115 days)	None reported	—	Completed planned course

It bears mentioning that no immunosuppressive factors were identified throughout the inpatient workup, with negative serologic testing for human immunodeficiency virus and viral hepatitis and a negative interferon-gamma release assay. Lymphocyte subset analysis, serum immunoglobulin levels, and complement assessment showed no evidence of immune dysfunction, and an autoimmune panel revealed no abnormalities. No additional advanced testing was performed.

The patient benefited from a multidisciplinary approach involving infectious diseases, maxillofacial surgery, and dermatology departments. The dermatology department assessed the patient in the emergency department and later performed a skin biopsy for histopathological evaluation, which was compatible with atypical mycobacteriosis. The infectious diseases department managed the overall clinical course, including antimicrobial selection, therapeutic drug monitoring, and toxicity management. The maxillofacial surgery department was actively involved throughout the hospitalization, providing regular assessments, guiding wound care, and collaborating closely in determining the need for further surgical debridement to optimize infection control.

**Figure 5:**
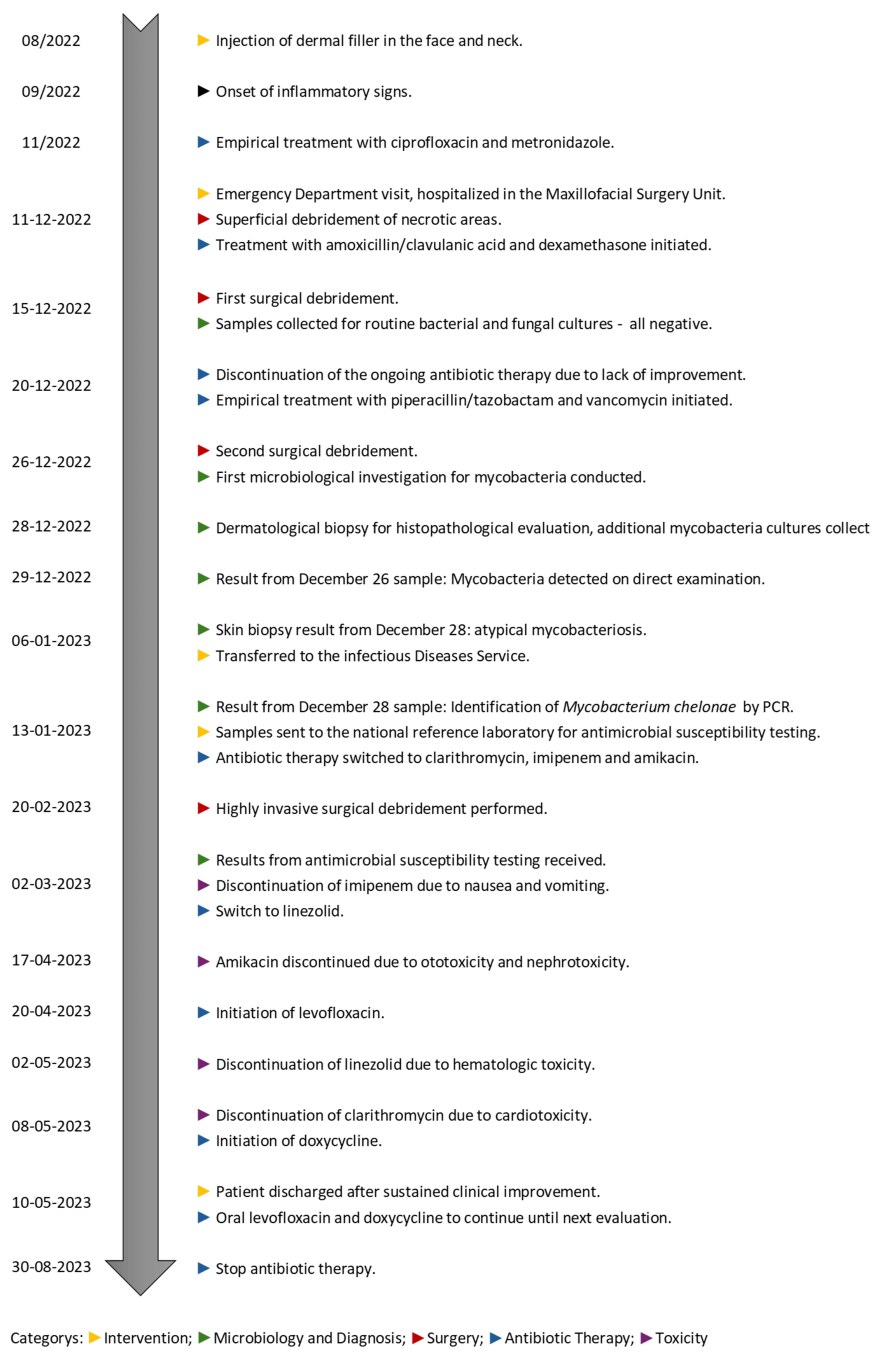
Timeline of the case report PCR: polymerase chain reaction

**Figure 6:**
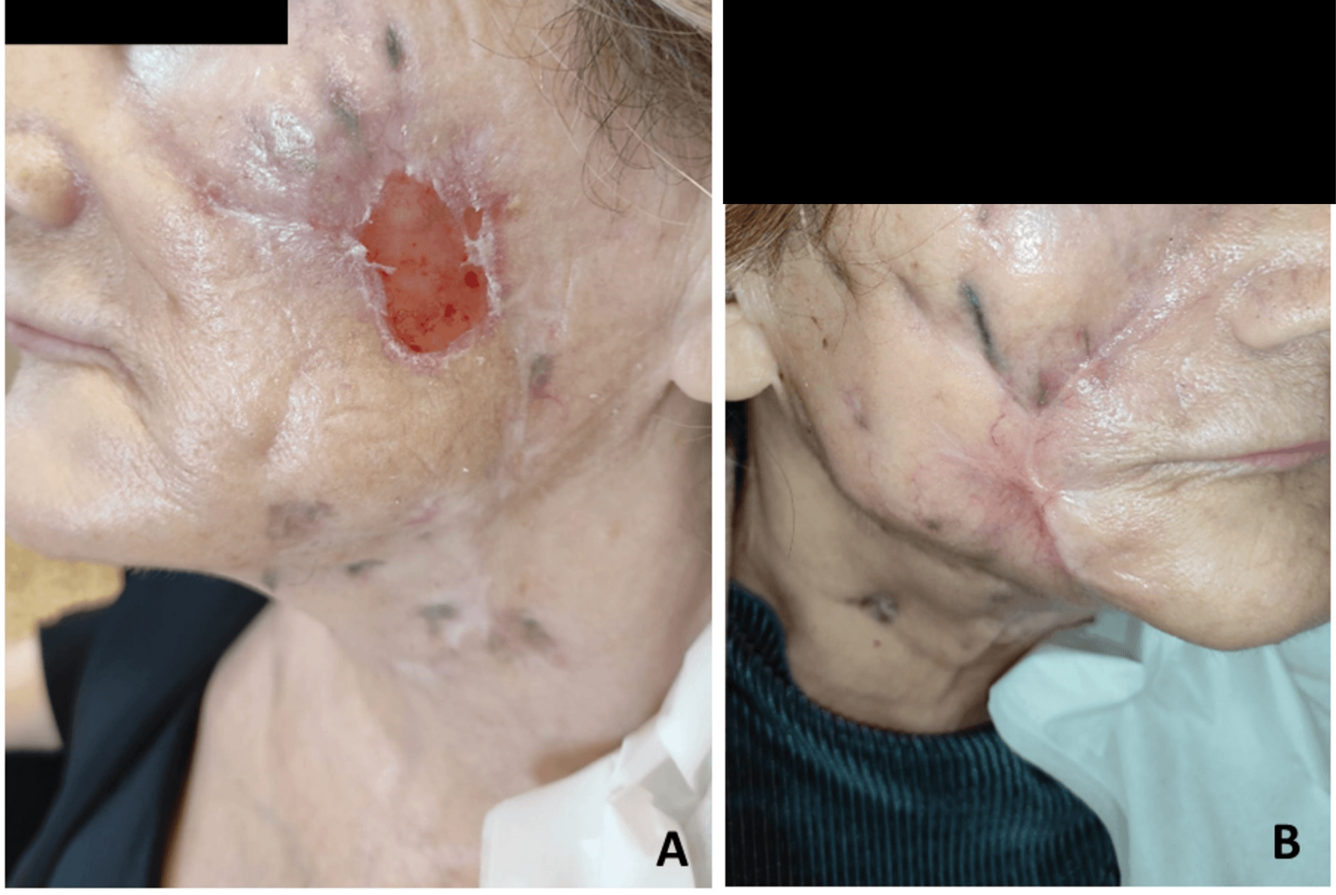
Skin lesions at the four-month post-discharge evaluation: (A) left side of the face and (B) right side of the face

Following sustained clinical improvement, the patient was discharged in early May 2023 with levofloxacin 500 mg every 24 hours and doxycycline 100 mg every 12 hours. For a more comprehensive understanding of the case’s chronological evolution, a timeline is presented in Figure 5. Four months after discharge, clinical evaluation showed significant improvement in the lesions observed, as depicted in Figure 6.

At a follow-up appointment in late August 2023, oral antibiotic therapy was discontinued after the resolution of all lesions and the absence of new lesions. The patient began facial rehabilitation physiotherapy and continued follow-up with the dermatology department, considering fractional CO2 laser treatment and maxillofacial surgery, which deemed skin grafts unnecessary. Otorhinolaryngology follow-up included evaluation for balance disturbance and sensorineural hearing loss secondary to aminoglycosides. The infectious diseases department follow-up also continued.

**Figure 7:**
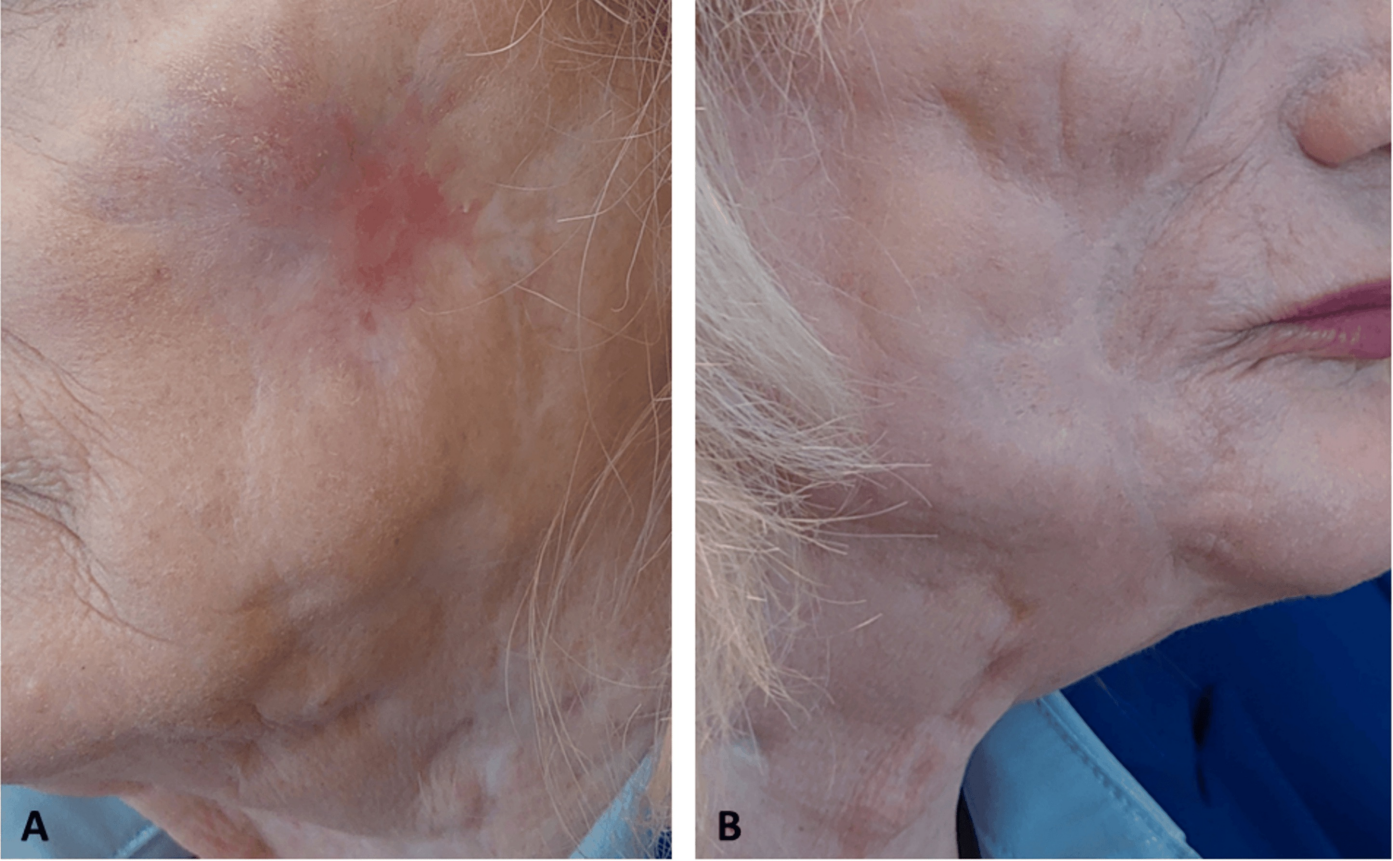
Full recovery at the 18-month post-discharge evaluation: (A) left side of the face and (B) right side of the face

At an 18-month post-discharge evaluation, the patient showed resolution of hyperalgesia and favorable evolution of mucosal fibrosis. There were no new nodular or exudative lesions, and the face was fully re-epithelialized, demonstrating excellent progress. Functional assessment revealed preserved facial nerve integrity, symmetrical facial expressions, and adequate oral competence. The patient maintained a full range of motion of the perioral and malar regions without significant contractures or functional impairment, allowing for normal speech and mastication (Figure 7).

## Discussion

*Mycobacterium chelonae* is a rapidly growing NTM increasingly recognized as a significant cause of cutaneous and soft-tissue infections, particularly following cosmetic procedures [2,7,9]. While primarily affecting immunocompromised individuals, immunocompetent patients can also develop *Mycobacterium chelonae* infections after skin trauma [2,10].

Preventing *Mycobacterium chelonae* infections following cosmetic procedures requires stringent adherence to infection control measures. One critical aspect is the proper sterilization and handling of injectable materials. For instance, the U.S. The Food and Drug Administration provides specific guidelines on preparing and administering poly-L-lactic acid fillers, emphasizing sterile diluents, aseptic reconstitution techniques, and single-use syringes to prevent contamination [5]. Any deviation from these protocols, including exposure to non-sterile water sources, increases the risk of introducing environmental mycobacteria into soft tissues [2,7,8].

Additionally, patient screening is essential in identifying individuals at higher risk of infectious complications. Patients with recent exposure to high-risk environments, such as non-medical cosmetic procedures, tattoo parlors, or mesotherapy, presenting tender, erythematous, draining nodules, chronic, nonhealing cellulitis, or skin ulcers, warrant thorough clinical assessment [2]. Similarly, individuals with a history of medical tourism should be carefully evaluated, as suboptimal infection control practices in certain regions have been associated with outbreaks of nontuberculous mycobacteria infections [8].

In this case, the patient developed a severe infection following a poly-L-lactic acid injection, emphasizing the potential risks associated with even seemingly minor cosmetic procedures in immunocompetent individuals. The initial empirical treatments with ciprofloxacin and metronidazole proved ineffective, aligning with the known resistance patterns of *Mycobacterium chelonae* [11,12].

The incidence of infections caused by *Mycobacterium chelonae* has increased in recent years, but accurate diagnosis continues to be challenging due to the variability in clinical presentations. As optimal treatment strategies have not yet been clearly established, it is essential to customize antibiotic therapy based on in vitro susceptibility testing for each case. Standard antibiotic regimens are often inadequate, which may lead to prolonged symptoms and complications [1].

Diagnosing *Mycobacterium chelonae* infections of the skin and soft tissue requires high clinical suspicion, culturing the bacteria from tissue biopsies and drainage materials, and conducting routine histopathologic analysis [2,10].

The delay in appropriate diagnosis and treatment likely contributed to the infection's progression and severity, highlighting the critical need for early consideration of rapidly growing nontuberculous infections in patients presenting with persistent inflammatory signs after cosmetic interventions.

Managing *Mycobacterium chelonae* infections typically involves a multifaceted approach combining surgical debridement and prolonged antibiotic therapy [2,7,9]. In this case, multiple surgical interventions, including extensive debridement, were necessary to control the infection's spread. The literature review and the severity of the lesions guided antibiotic selection. Prolonged antibiotic therapy, however, led to significant side effects, including cardiotoxicity, ototoxicity, and nephrotoxicity, emphasizing the importance of close patient monitoring and careful treatment regimen adjustments.

Treatment of *Mycobacterium chelonae* infections presents significant challenges, as it is one of the most resistant pathogenic rapidly growing mycobacteria. Established treatment guidelines are currently lacking, with most recommendations based on expert opinion [7,9,10].

While *Mycobacterium chelonae* is generally susceptible to macrolides, rapid resistance development can occur with monotherapy. Therefore, a multidrug regimen is typically recommended [2,7,11].

In vitro data suggest tobramycin as the most active aminoglycoside against *Mycobacterium chelonae*. Other antimicrobial agents with varying degrees of susceptibility include linezolid (54-90%), imipenem (40-60%), amikacin (50-70%), doxycycline (25%), and ciprofloxacin (20%). The backbone of treatment usually consists of a two- to three-drug regimen, typically including a macrolide and other agents based on disease extent [11,12]. First-line antitubercular agents (isoniazid, rifampin, pyrazinamide, and ethambutol) are ineffective against *Mycobacterium chelonae* [13].

Treatment duration varies depending on the infection site and severity. Severe skin and soft tissue infections generally require a minimum of four months of therapy, while bone infections may need six months or longer [11]. In this case, the antibiotic therapy duration was individualized and adjusted according to the patient's clinical evolution, response to treatment, and drug tolerance. Continuous clinical monitoring, including assessment of lesions, laboratory tests, and imaging studies, was crucial for evaluating treatment response and adjusting therapy as needed.

Empiric antibiotic therapy is often necessary while awaiting susceptibility results, especially in immunocompromised patients or those with severe infections, as observed in this case. Susceptibility testing for *Mycobacterium chelonae* can be time-consuming, with results in this case taking approximately six weeks. Prompt initiation of appropriate antibiotic therapy is crucial due to the high mortality in immunosuppressed patients, *Mycobacterium chelonae*'s resistance profile, and the potential for rapid disease progression and dissemination [11].

The successful resolution of the infection and the patient's eventual recovery demonstrate the effectiveness of a multidisciplinary approach involving infectious diseases specialists, dermatologists, maxillofacial surgeons, and other healthcare professionals. This case underscores the importance of patient education regarding the potential risks of cosmetic procedures and the need for prompt medical attention should complications arise.

To contextualize this case within the existing literature, a comparative analysis was performed between the present case, the first reported *Mycobacterium chelonae* infection following dermal filler injection in Europe, and the only other documented cases. These include a North American report describing a cluster of three cases [3] and a South Korean study reviewing 29 nontuberculous mycobacterial infections [14], of which only one was caused by *Mycobacterium chelonae* following dermal filler injection (Table 2).

**Table 2:** Comparative analysis of reported Mycobacterium chelonae infections following dermal filler injections PCR: polymerase chain reaction

Parameter	Present case (Europe)	North American cases [3]	Asian case [14]
Patient demographics	72-year-old immunocompetent woman	Cluster of three immunocompetent women (46–54 years)	50-year-old immunocompetent woman
Cosmetic procedure	Poly-L-lactic acid filler	Hyaluronic acid filler	Hyaluronic acid filler
Onset of symptoms	Delayed onset (weeks after procedure)	Rapid onset (within days to weeks)	Gradual onset (weeks after procedure)
Clinical presentation	Severe necrotic lesions on face and neck, deep ulcers and nodules with purulent drainage	Facial nodules, erythema, and minor abscesses	Single subcutaneous nodule on the cheek
Initial diagnosis	Misdiagnosed as cellulitis/foreign body reaction	Early suspicion of atypical infection	Initially diagnosed as bacterial abscess
Diagnostic delay	~4 months before correct identification	~3–6 weeks before culture results	~6 weeks before confirmation
Microbiological confirmation	Culture and PCR from biopsy samples	Culture from aspirate and wound specimens	Culture from biopsy sample
Empirical treatment	Ciprofloxacin, metronidazole, then broad-spectrum antibiotics	Clarithromycin and moxifloxacin	Clarithromycin and fluoroquinolones
Final antimicrobial therapy	Clarithromycin, amikacin, imipenem, linezolid	Clarithromycin, amikacin, linezolid	Clarithromycin and amikacin
Surgical interventions	Three major surgical debridements	No surgical intervention	No surgical intervention
Adverse effects	Cardiotoxicity, ototoxicity, nephrotoxicity, and hematologic toxicity from prolonged therapy	Hematologic toxicity due to linezolid	Mild gastrointestinal discomfort
Treatment duration	~7.5 months	~6 months of antibiotic therapy	~4 months of antibiotic therapy
Outcome	Complete resolution with residual fibrosis and scarring	Resolution without scarring	Resolution without scarring

While some differences in antimicrobial susceptibility and treatment approaches have been observed across reported cases, the limited sample size prevents definitive conclusions regarding regional variations. In Europe, the present case demonstrated susceptibility to clarithromycin, amikacin, linezolid, and moxifloxacin, with intermediate susceptibility to ciprofloxacin and resistance to sulfamethoxazole. This pattern aligns with previous European reports, where macrolides remain effective but require combination therapy to prevent resistance [2,7,11]. In North America, cases were treated with clarithromycin, amikacin, and linezolid; however, notable fluoroquinolone resistance was observed. These infections were linked to contaminated tap water, highlighting environmental exposure as a potential risk factor [3]. In Asia, the only documented case showed susceptibility to clarithromycin and amikacin, with fluoroquinolone use also reported, and treatment duration in this case was relatively shorter [14]. Given the small number of reported cases, further large-scale studies are needed to establish meaningful regional trends in antimicrobial resistance and treatment responses.

## Conclusions

This case report illustrates the complexity of managing *Mycobacterium chelonae* infections following cosmetic procedures. The patient's prolonged course of illness, multiple surgical interventions, and extensive antibiotic therapy underscore the challenges in diagnosing and treating NTM infections. Early recognition and appropriate management, facilitated by a multidisciplinary approach, are crucial to prevent severe complications and improve patient outcomes. Diagnostic methods encompass clinical assessments, microbiological and molecular tests, drug susceptibility testing, and imaging studies. Treatment typically involves a combination of antibiotics, with macrolides being the backbone drug.

Furthermore, this case highlights the potential for severe complications arising from seemingly minor cosmetic procedures, even in immunocompetent individuals. Given the increasing incidence of *Mycobacterium chelonae* infections linked to aesthetic interventions, heightened awareness among healthcare providers and patients is crucial. Strict adherence to infection control measures, including sterilization protocols, aseptic techniques, and thorough patient screening, is essential to minimize infection risk. A high index of suspicion and prompt medical attention are key to managing complications and ensuring patient safety following cosmetic procedures.
